# Impact of the COVID-19 Pandemic on Surgical Education and Training: A Resident Survey in a Developing Country

**DOI:** 10.7759/cureus.45283

**Published:** 2023-09-15

**Authors:** Muhammad Salman Shafique, Muhammad Arham, Sayyam Fatima, Hamza Waqar Bhatti

**Affiliations:** 1 General Surgery, Chesterfield Royal Hospital, Chesterfield, GBR; 2 General Surgery, Rawalpindi Medical University, Rawalpindi, PAK; 3 Surgical Unit I, Holy Family Hospital, Rawalpindi, PAK; 4 Surgical Unit II, Benazir Bhutto Hospital, Rawalpindi, PAK

**Keywords:** psychological well-being, surgery residents, surgical training, surgical education, covid-19

## Abstract

Background

The COVID-19 pandemic has adversely affected medical education and training programs worldwide. Early investigations have shown that surgical residents face a lot of challenges in these unprecedented times. This study aims to assess the impact of the COVID-19 pandemic on surgical education and training in a developing country.

Methods

This cross-sectional study was conducted in Allied Hospitals of Rawalpindi Medical University, Rawalpindi, Pakistan from June 2021 to July 2021. A structured questionnaire designed by the researchers was distributed to all surgery departments, and surgical residents who consented to participate in this study were included.

Results

A total of 152 residents participated in this study, of which 53 (34.9%) were in general surgery and 99 (65.1%) in various surgical allied specialties. Of the residents, 14.5% reported full transfer from the parent unit to the COVID-19 unit. An increase in emergency surgical procedures was reported by 52.8% of general surgery residents as compared to surgical allied specialties (P = 0.037). Of the residents, 90.1% reported increased stress and anxiety levels, with the number of allied residents significantly higher than general surgery residents (P = 0.031). A total of 125 (82.2%) respondents claimed that fear of contracting the virus affected proper patient evaluation.

Conclusion

The COVID-19 pandemic has severely impacted the training and psychological well-being of surgical residents.

## Introduction

The coronavirus disease 2019 (COVID-19) infection, caused by SARS-CoV-2, initially originated in Wuhan province of China and is now a global pandemic [1]. This pandemic has significantly affected the healthcare system, which already faces a lot of challenges in developing countries like Pakistan [2]. Due to its rapid spread, healthcare authorities around the globe introduced necessary policies regarding social distancing and took drastic measures of reallocation of medical services [3]. To control the spread and reduce the burden of the virus, one of the measures was the cancellation of elective procedures and outpatient clinical care to free the workforce for the care of COVID-19 patients and potentially flatten the curve [4]. The limitation of procedural training and outpatient care has fairly affected medical education and training in all specialties, particularly surgical fields.

Surgical education and training are predominantly affected because outpatient management and elective procedural skills are principal components of core surgical training and are necessary for surgeons to provide optimum clinical care [3,4]. Moreover, with rapid advancements in clinical information, education, and technology, it is imperative to master these surgical skills for independent practice and to train future surgeons [5]. All these factors are extremely necessary for the professional growth of a surgeon and the current pandemic has had a negative influence on surgeons’ education and professional growth [6].

Similarly, surgical training courses, conferences, electives, and examinations are either delayed or canceled due to the pandemic [7]. To reduce the risk of peri-operative spread of SARS-CoV-2 infection, all elective procedures had to be canceled and only emergency surgeries were performed [8]. Moreover, to decrease the length of patients’ stay in hospitals and its associated risk of increased viral transmission, most of the emergencies were managed conservatively [6]. Furthermore, policies of minimizing the staff in the operating room inadvertently limited the hands-on learning opportunities for surgical residents of various specialties [7]. These drastic changes have resulted in a severe reduction of learning and training opportunities for surgical trainees.

Although surgical education has been affected worldwide, developing countries suffered the most due to their limited resources and poor surgical training infrastructure [2,9]. Thus, it is important to evaluate the impact of COVID-19 on surgical training along with resident surgeons’ perceptions, especially in a developing country like Pakistan. This study aims to evaluate and quantify the impact of COVID-19 on surgical training and education to ascertain the adverse effects of deficient learning opportunities. Moreover, our study also aims to provide recommendations to alleviate the ongoing challenges. This will aid us in exploring different ways to adapt and maintain a consistent learning environment in subsequent COVID-19 waves.

This article was previously presented as a poster at “The Association of Surgeons in Training (ASIT) Annual Conference 2022 - Surgical Training: The Evolution” on March 6, 2022.

## Materials and methods

This descriptive cross-sectional study included 152 residents of surgery and allied specialties. It was conducted from June 2021 to July 2021. A structured questionnaire designed by the researchers was distributed to all surgery departments, and surgical residents who consented to participate in this study were included. Ethical permission was obtained from the Research & Ethical Committee of Rawalpindi Medical University & Allied Hospitals, Rawalpindi (Ref. No.: 138/IREF/RMU/2021). All surgical residents responded to the survey, making a response rate of 100%. All questionnaires were filled completely by the respondents.

The questionnaire consisted of a total of 30 items, divided into various domains that assessed surgical residents’ perception regarding the impact of the COVID-19 pandemic on surgical training and education. It included demographic details, the impact of the pandemic on surgical, clinical, academic, and research activities, the impact on residents’ future careers, and psychological well-being. The validity of the questionnaire was assessed using Cronbach’s alpha. Data were analyzed using IBM SPSS version 25 (IBM Corp., Armonk, NY). Frequencies and percentages of each response of the included participants were evaluated. The difference and association of the received responses between residents of general surgery and surgical allied specialties were assessed using Pearson’s chi-square test. A p-value of 0.05 or less was considered statistically significant.

## Results

A total of 152 residents participated in this study with 61 (40.1%) males and 91 (59.9%) females. The mean age of the respondents was 28.4 ± 1.8 years. Of these 152 respondents, 53 (34.9%) were training in general surgery, whereas the remaining 99 (65.1%) were enrolled in various surgical allied specialties. Only a few residents (7, 4.6%) had an underlying comorbid condition. These findings are depicted in Table 1.

**Table 1:** Sociodemographic characteristics of respondents

Characteristics	Frequency (%)
Total	152 (100%)
Gender	
Male	61 (40.1%)
Female	91 (59.9%)
Specialty	
General surgery	53 (34.9%)
Obstetrics and gynecology	24 (15.8%)
Otolaryngology	19 (12.5%)
Orthopedic surgery	14 (9.2%)
Ophthalmology	12 (7.9%)
Urology	10 (6.6%)
Plastic surgery	08 (5.3%)
Pediatric surgery	07 (4.6%)
Neurosurgery	05 (3.3%)
Year of residency	
First	30 (19.7%)
Second	52 (34.2%)
Third	41 (27.0%)
Fourth	29 (19.1%)
Comorbid condition	
Yes	07 (4.6%)
No	145 (95.4%)

**Figure 1:**
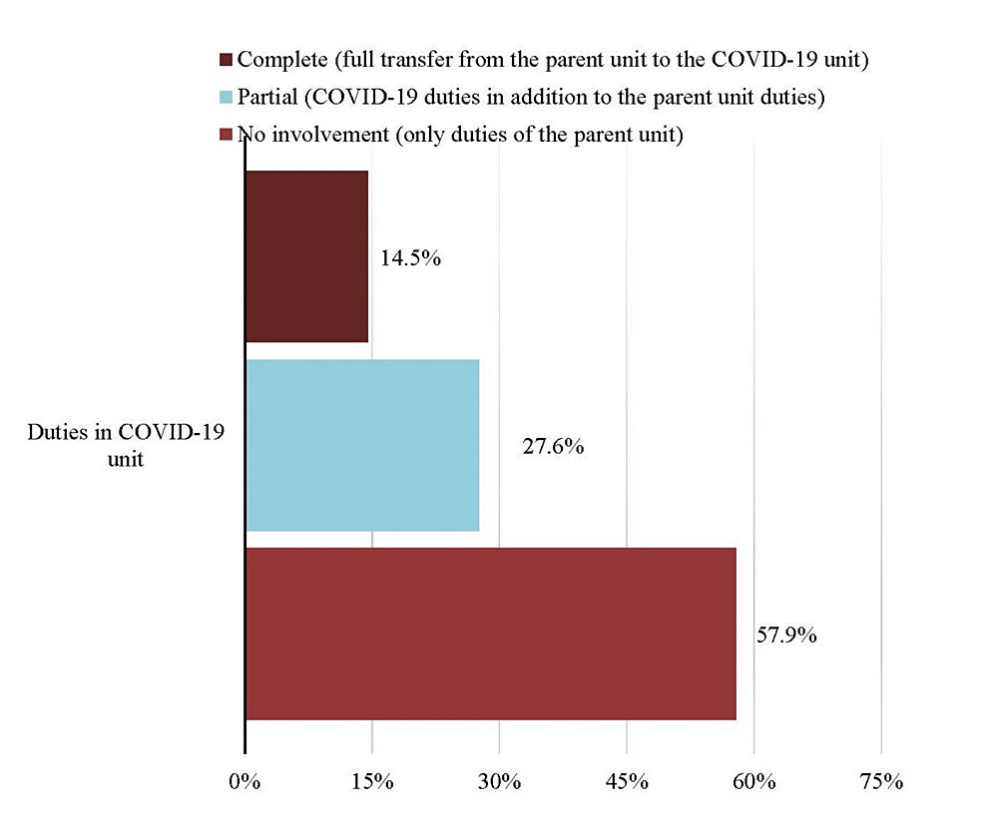
Graphical representation of respondents according to duties assigned in the COVID-19 unit

Many residents reported full transfer from the parent unit to the COVID-19 unit (22, 14.5%) or COVID-19 duties in addition to parent unit duties (42, 27.6%), as illustrated in Figure 1.

Only 10 (6.6%) residents voluntarily took part in a COVID-19 emergency unit to help fill the shortage of frontline healthcare workers’ force. Many residents reported a significant reduction in surgical activities but with one or more weekly planned surgical sessions (59, 38.8%) or a significant reduction with less than one surgical session per week (23, 15.1%). Many respondents reported that they have either decreased their usual research activities (52, 34.2%) or completely interrupted them (33, 21.7%) because of the COVID-19 pandemic. Table 2 highlights these findings.

**Table 2:** Impact of the COVID-19 pandemic on clinical, surgical, and research activities

Characteristics	Frequency (%)
Impact of the COVID-19 pandemic on clinical activities	
I maintained my usual activity	93 (61.2%)
I have been moved to a non-surgical unit (e.g., COVID ward, internal medicine ward, and emergency)	29 (19.1%)
I voluntarily took part in a COVID-19 emergency unit	10 (6.6%)
I interrupted all clinical activities beforehand	03 (2.0%)
I interrupted all clinical activities because I started feeling COVID-related symptoms (temporary)	15 (9.9%)
I interrupted all clinical activities because I started feeling COVID-related symptoms (permanent)	02 (1.3%)
Impact of the COVID-19 pandemic on surgical activities	
No relevant modifications	41 (27.0%)
A significant reduction but with one or more weekly planned surgical session	59 (38.8%)
A significant reduction with less than one surgical session per week	23 (15.1%)
Complete interruption of all surgical activities	26 (17.1%)
Increase in surgical activity	03 (2.0%)
Impact of the COVID-19 pandemic on research activities	
I maintained my usual activity	53 (34.9%)
I increased my usual activity	07 (4.6%)
I decreased my usual activity	52 (34.2%)
I interrupted my usual activity	33 (21.7%)
I usually do not perform this kind of activity	07 (4.6%)

A vast majority of the residents (127, 83.6%) reported that the COVID-19 pandemic had a negative impact on their clinical training program. Many also believed that the pandemic has significantly changed their future job ambitions (62, 40.8%), as shown in Table 3.

**Table 3:** Impact of COVID-19 on clinical training program and residents’ future career

Characteristics	Frequency (%)
Impact of the COVID-19 pandemic on clinical training program	
I believe it had a negative impact	127 (83.6%)
I believe it had no significant impact	25 (16.4%)
I believe it had a positive impact	0 (0.0%)
Impact of the COVID-19 pandemic on resident’s future career	
It has significantly changed my future job ambitions	62 (40.8%)
I believe it may have broadened my future job opportunities	21 (13.8%
None	69 (45.4%)

**Figure 2:**
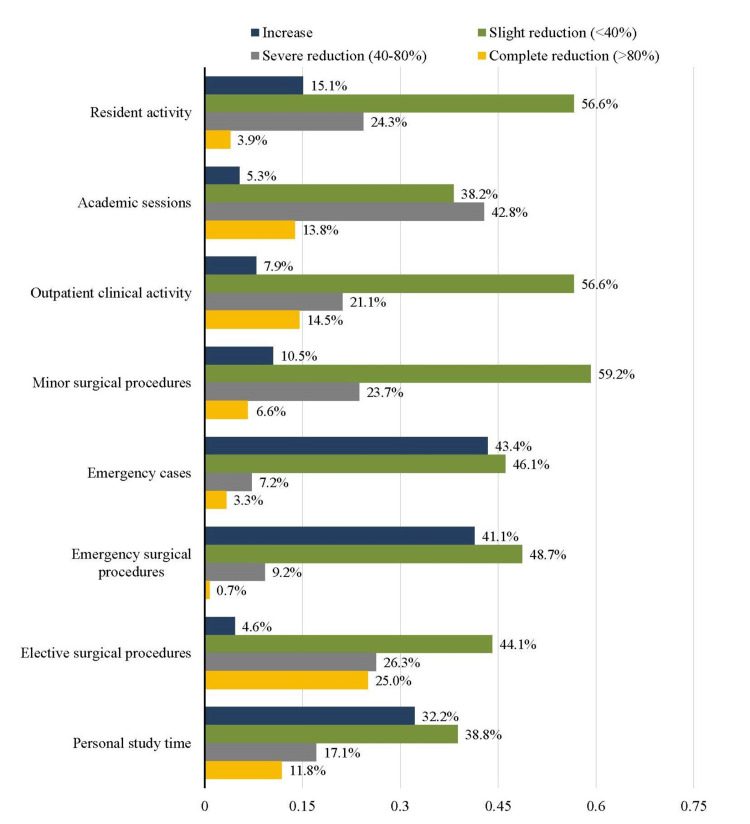
Impact of the COVID-19 pandemic on clinical experience of residents

More than half of the residents reported a severe to complete reduction of academic sessions (86, 56.6%) and elective surgical procedures (78, 51.3%). A total of 86 (56.6%) respondents reported a slight reduction of outpatient clinical activity, whereas 54 (35.6%) residents reported a severe to complete reduction, as illustrated in Figure 2.

However, many residents reported an increase in the number of emergency cases (66, 43.4%) and emergency surgical procedures (63, 41.4%), with the number of general surgery residents being significantly higher than residents of other specialties in both groups (p-value of 0.006 and 0.037, respectively), as depicted in Table 4.

**Table 4:** Impact of the COVID-19 pandemic on emergency cases and surgical procedures according to specialty * Chi-square test applied.

		Increase, n (%)	Decrease, n (%)	P-value*
Emergency cases	General surgery	31 (58.5%)	22 (41.5%)	0.006
	Surgical allied	35 (35.4%)	64 (64.6%)	
Emergency surgical procedures	General surgery	28 (52.8%)	25 (47.2%)	0.037
	Surgical allied	35 (35.4%)	64 (64.6%)	

Of the surgical residents, 85.5% believed that the loss of surgical training opportunities during the pandemic would negatively impact their job performance, and 28.3% of residents suggested an extension in the training period. More than three-fourths (77.6%) of the residents had to cancel their participation in workshops, conferences, or symposia. More than 50% of the respondents reported that their hospital did not offer complementary virtual tutoring for its residents. Of the residents, 46.7% reported the introduction of hospital-based training programs for the surgical teams regarding the management of surgical patients with suspected or confirmed COVID-19 infection. Similarly, 69.1% of respondents reported the implementation of protocols for the surgical care of patients with suspected or confirmed COVID-19 infection. These findings are depicted in Table 5.

**Table 5:** Residents’ perception and hospital policy during the COVID-19 pandemic

Question	Yes, n (%)	No, n (%)	Uncertain, n (%)
Loss of surgical training opportunities during the COVID-19 pandemic will negatively impact your job performance	130 (85.5%)	14 (9.2%)	08 (5.3%)
Had to cancel your participation in events? (Workshops, Conferences, Symposia)	118 (77.6%)	32 (21.1%)	02 (1.3%)
Has your hospital implemented any protocol for surgical care of patients with suspected or confirmed COVID-19 infection?	105 (69.1%)	31 (20.4%)	16 (10.5%)
Has your hospital introduced any training program for the surgical team regarding the management of suspected or confirmed surgical patients with COVID-19?	71 (46.7%)	59 (38.8%)	22 (14.5%)
Has your hospital implemented complementary virtual tutoring for surgical residents?	41 (27.0%)	82 (53.9%)	29 (19.1%)
Do you suggest extending training after graduation?	43 (28.3%)	99 (65.1%)	10 (6.6%)

**Figure 3:**
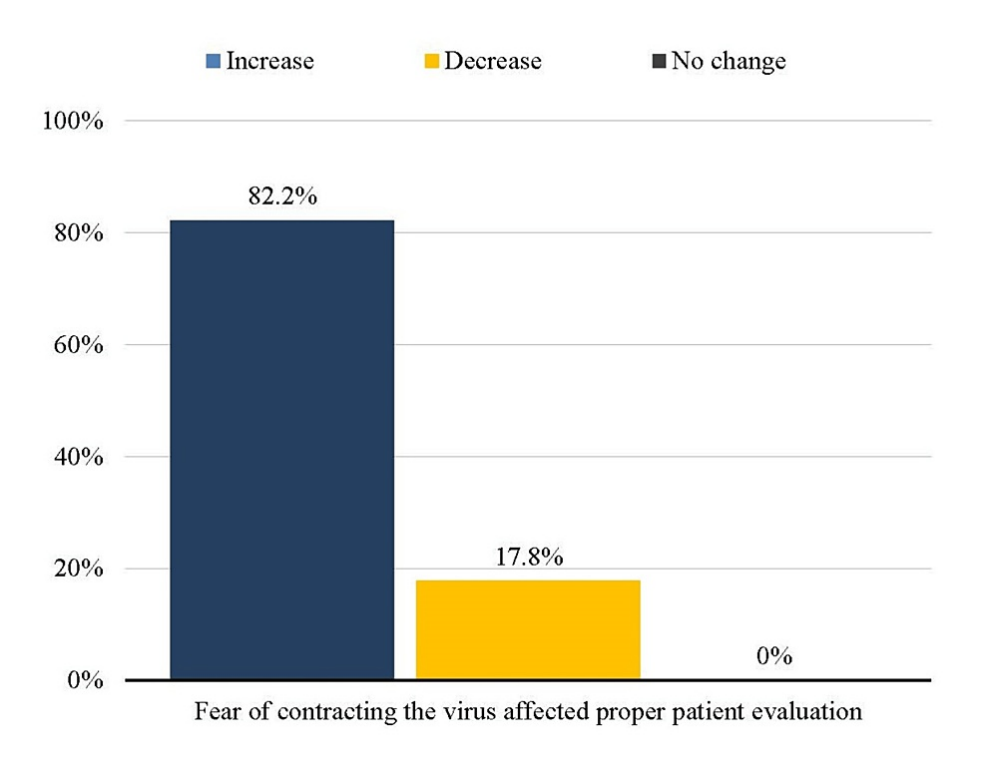
Impact of fear of contracting the virus on proper patient evaluation

Of the residents, 137 (90.1%) reported an increase in their stress and/or anxiety levels due to the pandemic with the number of surgical allied residents being significantly higher as compared to general surgery residents (P = 0.031). A vast majority reported fear of contracting the virus at work (133, 87.5%) and that this fear affected proper patient evaluation (125, 82.2%), as shown in Figure 3.

**Figure 4:**
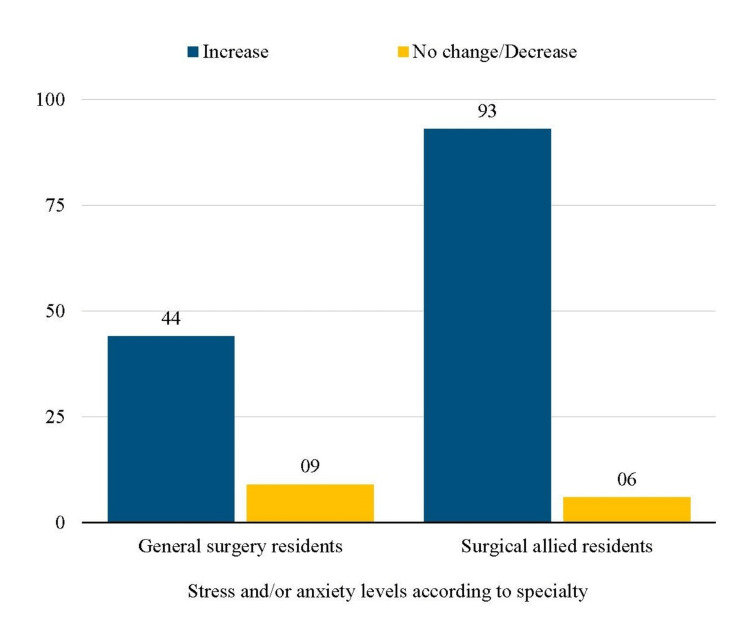
Number of residents reporting stress/anxiety due to the COVID-19 pandemic

Nearly all residents (150, 98.7%) reported increased fear of transmitting the virus to family members. Stress and anxiety levels were found to be higher among surgical allied residents as compared to the general surgical residents (Figure 4).

## Discussion

The COVID-19 pandemic has affected medical education and training worldwide, with surgical specialties being impacted the most [3,10]. To contain the spread of the virus, unprecedented measures like social distancing and lockdowns were introduced. Since in-hospital viral transmission was considered a grave threat, outpatient services and elective surgeries were canceled or postponed [2]. The American College of Surgeons also issued guidelines to postpone most non-emergent elective surgical procedures [11]. Many countries, including developing nations, adopted these practices to tackle the COVID-19 emergency and many investigations from the early phase of the pandemic reported decreased outpatient clinic consultations and elective surgical cases and its associated negative impact on the training of surgical residents [2,12]. Our study was conducted one year after the virus was first detected in Pakistan and shows that this COVID-19 pandemic continues to affect surgical education. More than half of the respondents in our study reported a severe to complete reduction of elective surgical procedures and a vast majority also reported seeing fewer patients in the outpatient clinics, limiting their learning opportunities. However, many residents in our study reported increased emergency cases, which could be due to a backlog of outpatient consultations presented in the emergency.

Across the globe, increasing COVID-19 admissions overwhelmed hospitals’ capacity, and many surgical residents were shifted to COVID-19 units to fill in the shortage of frontline healthcare workers [3,10]. In our study, 42.1% of surgical residents were assigned duties to manage COVID-19 patients and were therefore spending less time in their surgical training program. Only 6.6% of the respondents voluntarily took part in a COVID-19 emergency unit, showing that hospital policy compelled surgical residents to assume these new roles. Considering these hurdles in surgical education, many programs in the developed world modified their curricula to include online didactics and virtual learning tools to augment the training of surgical residents [13,14]. These included online case discussions, virtual academic sessions, and video-based education. However, the majority of respondents in our study reported that their hospital was not offering any virtual tutoring platform for the residents, possibly because of a lack of required infrastructure and internet connectivity in public hospitals. In addition to virtual learning, many centers advocated the increased use of surgical simulators for trainees since research has proven the efficacy of simulators in enhancing surgical skills [15]. In times, when surgical residents are less exposed to hands-on learning opportunities, employing these additional tools of teaching is of paramount importance. Although they cannot substitute for the actual learning experience, their significance in these challenging times cannot be ignored. Hospitals in developing countries should promote the utilization of these new learning modalities by ensuring adequate provision of the appropriate hardware, equipment, and stable internet access for surgical trainees.

Apart from significantly minimizing clinical and surgical learning opportunities, the COVID-19 pandemic also had an impact on the academic and research activities of surgical trainees. More than half of the trainees in our study reported a severe to complete reduction in academic sessions. Many residents either decreased or completely interrupted their research activities. This contrasts with reports from other programs around the world, where decreased surgical case volume meant that residents had more time to pursue research projects [12,16-18]. In addition, surgical residents reported increased time for personal study during the COVID-19 pandemic [2], whereas only 32.2% of our respondents reported having more time to study. This is understandable, considering the increased workload on surgical residents who were assigned duties to manage COVID-19 patients in addition to surgical responsibilities.

Hence, nearly all aspects of surgical training were impacted by the pandemic, and the majority of the residents in our survey reported that this COVID-19 emergency had a negative impact on their surgical training program, with many also believing that the loss of surgical training opportunities will disrupt their job performance. Similar resident opinions were also documented in other surveys [2,12,14]. These drastic changes imposed by the pandemic were not without its psychological effects. Of our residents, 137 (90.1%) reported increased stress and/or anxiety due to this new grave situation, as mirrored by investigations of other authors around the globe [8]. The majority of trainees in our survey cited concerns about contracting the virus at work and believed that this fear affected their ability to evaluate patients properly. Similar views were also held by surgical residents of Nigeria [2]. Shortages of personal protective equipment (PPE) have been identified as a factor negatively influencing trainee well-being and could be a possible explanation for these fears [19]. Hospitals should ensure an adequate supply of protective resources for all residents to mitigate these concerns, which inadvertently affect patient care.

There are some limitations to this study. Apart from the general limitations of a survey-based study, our study required respondents to compare all parameters of their training with the pre-pandemic situation, which raises the possibility of recall bias. Moreover, our study was conducted in only three public hospitals affiliated with the same institution, hence limiting the generalizability of the results.

## Conclusions

This study concluded that the COVID-19 pandemic has adversely impacted all aspects of surgical education and training in a developing country. This pandemic has severely limited the surgical, clinical, academic, and research activities of surgical residents across all specialties. Overall, the pandemic has had a negative influence on the surgical training program and the psychological well-being of trainees.
